# Nitrogen and Iron Availability Drive Metabolic Remodeling and Natural Selection of Diverse Phytoplankton during Experimental Upwelling

**DOI:** 10.1128/msystems.00729-22

**Published:** 2022-08-29

**Authors:** B. C. Kolody, S. R. Smith, L. Zeigler Allen, J. P. McCrow, A. Moustafa, D. Shi, B. M. Hopkinson, F. M. M. Morel, B. B. Ward, A. E. Allen

**Affiliations:** a Scripps Institution of Oceanography, University of California, San Diego, California, USA; b Microbial and Environmental Genomics Group, J. Craig Venter Institute, La Jolla, California, USA; c Moss Landing Marine Laboratories, San José State University, Moss Landing, California, USA; d Department of Biology, American University in Cairo, New Cairo, Egypt; e State Key Laboratory of Marine Environmental Science, Xiamen University, Xiamen, Fujian, People’s Republic of China; f Department of Marine Sciences, University of Georgia, Athens, Georgia, USA; g Department of Geosciences, Princeton University, Princeton, New Jersey, USA; UiT—The Arctic University of Norway

**Keywords:** bloom, diatom, iron, metatranscriptome, nitrogen, phytoplankton

## Abstract

Nearly half of carbon fixation and primary production originates from marine phytoplankton, and much of it occurs in episodic blooms in upwelling regimes. Here, we simulated blooms limited by nitrogen and iron by incubating Monterey Bay surface waters with subnutricline waters and inorganic nutrients and measured the whole-community transcriptomic response during mid- and late-bloom conditions. Cell counts revealed that centric and pennate diatoms (largely Pseudo-nitzschia and Chaetoceros spp.) were the major blooming taxa, but dinoflagellates, prasinophytes, and prymnesiophytes also increased. Viral mRNA significantly increased in late bloom and likely played a role in the bloom’s demise. We observed conserved shifts in the genetic similarity of phytoplankton populations to cultivated strains, indicating adaptive population-level changes in community composition. Additionally, the density of single nucleotide variants (SNVs) declined in late-bloom samples for most taxa, indicating a loss of intraspecific diversity as a result of competition and a selective sweep of adaptive alleles. We noted differences between mid- and late-bloom metabolism and differential regulation of light-harvesting complexes (LHCs) under nutrient stress. While most LHCs are diminished under nutrient stress, we showed that diverse taxa upregulated specialized, energy-dissipating LHCs in low iron. We also suggest the relative expression of *NRT2* compared to the expression of *GSII* as a marker of cellular nitrogen status and the relative expression of iron starvation-induced protein genes (*ISIP1*, *ISIP2*, and *ISIP3*) compared to the expression of the thiamine biosynthesis gene (*thiC*) as a marker of iron status in natural diatom communities.

**IMPORTANCE** Iron and nitrogen are the nutrients that most commonly limit phytoplankton growth in the world’s oceans. The utilization of these resources by phytoplankton sets the biomass available to marine systems and is of particular interest in high-nutrient, low-chlorophyll (HNLC) coastal fisheries. Previous research has described the biogeography of phytoplankton in HNLC regions and the transcriptional responses of representative taxa to nutrient limitation. However, the differential transcriptional responses of whole phytoplankton communities to iron and nitrogen limitation has not been previously described, nor has the selective pressure that these competitive bloom environments exert on major players. In addition to describing changes in the physiology of diverse phytoplankton, we suggest practical indicators of cellular nitrogen and iron status for future monitoring.

## INTRODUCTION

Most phytoplankton growth in the world’s oceans is limited by either nitrogen (N) or iron (Fe) (1). Nitrogen is a macronutrient essential to the structure of nucleotides and amino acids (2), and iron is a micronutrient that is crucial for redox reactions, including photosynthesis and respiration (3). Despite sustaining substantial fisheries, the California Current is considered a high-nutrient, low-chlorophyll (HNLC) zone, meaning there is often insufficient iron for nitrogen to be utilized fully (4, 5). In such an environment, phytoplankton compete for not only the nitrogen necessary for growth, but also the iron required for photosynthesis and essential functions. During upwelling, iron-rich waters stimulate rapid phytoplankton growth. The community members that are able to successfully bloom not only structure the local ecology and biogeochemistry but may also have important human health impacts, e.g., large diatoms like Pseudo-nitzschia spp. that produce the toxin domoic acid (6) or dinoflagellates like Karenia brevis that produce brevetoxin (7). The duration and extent of these blooms are controlled top-down by predation and viral lysis, as well as bottom-up by depletion of essential nutrients (8).

Several factors contribute to a successful nutrient response. Small cells have greater uptake capacity relative to their nutritional needs, making them better competitors in oligotrophic regions (9, 10), whereas large cells have greater storage capacity. For example, dinoflagellates can store a large amount of N by accumulating amino acids (11, 12) and producing triacylglycerol (13), and harmful dinoflagellate blooms have often been associated with high nitrogen (11, 14, 15). Differences in nutrient responsiveness are also thought to contribute to the success of large diatoms following upwelling in HNLC regions (16, 17). Diatoms can upregulate nitrogen assimilation genes in response to the absence of fixed nitrogen, prior to the onset of nitrogen availability (18), can take up and store more nitrogen than is immediately needed (e.g., luxury uptake) (19), and have higher maximum nitrate uptake rates than dinoflagellates and chlorophytes (20). Diatoms also possess an ornithine-urea cycle (21) which may increase flexibility in nitrogen-replete conditions.

Motility is also an important factor because it allows phytoplankton to seek out nutrients. Pelagic diatoms, which are mostly nonmotile, thrive in turbulent environments (22), in contrast to many flagellated haptophytes (23), pelagophytes (24), cryptophytes (25), and dinoflagellates (26). Several dinoflagellate species can take up nitrogen in the dark and can perform diel vertical migrations to acquire nutrients from deeper waters (11).

Finally, the flexibility to take up nutrients from larger organic molecules can be helpful when inorganic pools dwindle. The pelagophyte Aureococcus anophagefferens dominates when inorganic N is low but dissolved organic nitrogen (DON) is high (27). Some dinoflagellates take this a step further to facultative mixotrophy; Ceratium furca (28), Prorocentrum minimum (29), and Gyrodinium galatheanum (30) become predatory when nitrate is limiting.

In the case of iron limitation, several taxa possess iron-free enzymatic alternatives, such as Ni- superoxide dismutases (SODs) in picoprymnesiophytes (31) and Mn^−^ SODs in diatoms (32). Diatoms can also preferentially uptake nitrogen species that do not require reduction by iron metalloproteins (32) and can partition newly available iron toward assimilating nitrate more rapidly than haptophytes and chlorophytes (32). While much effort has gone into understanding the success of diatoms in bloom situations, less is known about the bloom responses of other major lineages, including dinoflagellates, chlorophytes, haptophytes, and pelagophytes, which are also persistent members of the community (32). Additionally, the responses of major players to nitrogen (16, 33) and iron limitation (32, 34–36) are most commonly studied individually, and there is still much to learn about how the transcriptional dynamics of whole communities shift when moving from rapid proliferation to nutrient stress. Finally, very little is known about population-level genetic shifts in response to blooms and the selective pressure that nutrient limitation exerts on genes (37, 38).

Here, we compare the diversity and physiology of California Current phytoplankton communities responding to simulated blooms terminating with either nitrogen or iron limitation. In addition to characterizing their metabolic and nutrient acquisition strategies, we examine the role of population shifts and selection in blooms, in which algae compete for scarce nutrients. We consider the nitrogen and iron response of not only diatoms, which bloomed most dramatically, but also the entire eukaryotic phytoplankton community, including dinoflagellates, chlorophytes, haptophytes and pelagophytes. Finally, we consider the role of viruses in bloom demise.

## RESULTS AND DISCUSSION

### Community response to nitrogen depletion.

**FIG 1:**
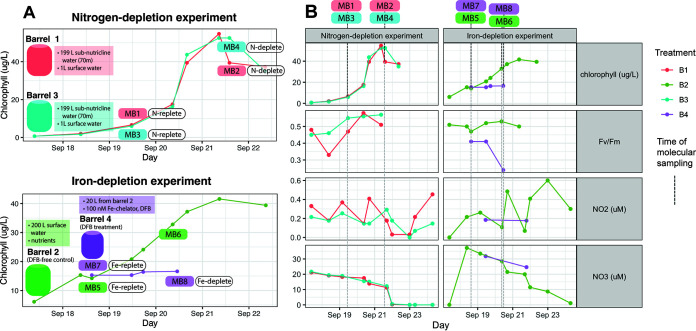
(A) Illustration of experimental design for nitrogen depletion (top) and iron depletion (bottom) experiments. The nitrogen depletion experiment consisted of incubation barrels B1 and B3 (red and blue), and the iron depletion experiment consisted of barrels B2 and B4 (green and purple). Timing of molecular samples (MB1 to -8) is shown superimposed over chlorophyll concentrations (*y* axis; colored by barrel). In the nitrogen depletion experiment, barrels 1 (red) and 3 (blue) were duplicates, both containing 199 L subnutricline water sampled from a 70-m depth and 1 L of surface water. In the iron depletion experiment, barrel 2 (green) contained 200 L of surface water with nutrients added to achieve final concentrations of 40 μM nitrate, 2.5 μM phosphate, and 50 μM silica. In this barrel, iron levels were left unmanipulated as a control. On September 18, barrel 4 (purple) was created by subsampling 20 L from barrel 2. In barrel 4, a low-iron environment was created with the addition of the Fe chelator DFB (final concentration, 100 nM). (B) Chlorophyll, *F_v_*/*F_m_* (variable fluorescence/maximum fluorescence), nitrite, and nitrate measurements across time for all experimental conditions (see SD2 at https://doi.org/10.5281/zenodo.6953574; barrels colored as described in the legend to panel A). Timing of molecular samples is given by dotted gray vertical lines.

**FIG 2:**
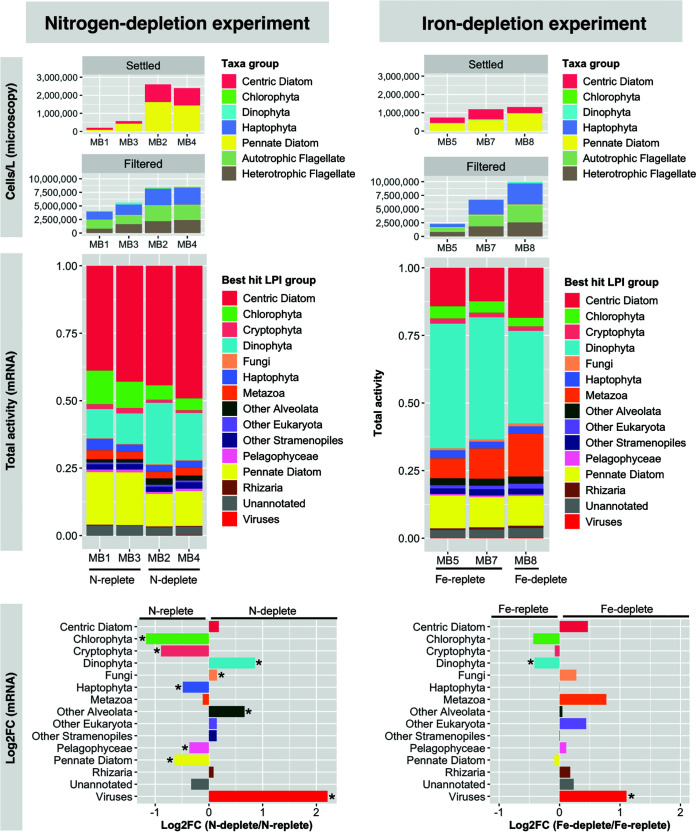
Coarse taxonomy across both experiments via cell counts (top), proportions of total mRNA (middle), and differential abundances of taxon group mRNA (bottom) across nutrient conditions. For microscopy (top), “settled” cells represent large diatoms and dinoflagellates that were separated via Utermöhl settling technique and “filtered” cells represent small taxa that could only be counted after collection on an 0.8-μm filter. For differential mRNA abundance (bottom), log_2_ fold changes of taxon group activities are depicted as proportions of library reads across nitrogen (left) and iron (right) conditions. Asterisks denote significant differences (edgeR, FDR < 0.05). MB6 was not included in the differential expression analysis because, although no iron chelator was added, it appeared to be iron limited due to being a late-stage bloom condition.

In the nitrogen depletion experiment, subnutricline water was combined with surface water in duplicate to induce a phytoplankton bloom (Fig. 1 and 2). Indeed, chlorophyll rose from <1 μg/L at the time of inoculation to >50 μg/L at the peak of the bloom (Fig. 1; see data set SD2B at https://doi.org/10.5281/zenodo.6953574) and then dropped as nitrate was drawn down (Fig. 1B; see SD2A at the DOI mentioned above). A complete drawdown of nitrate, along with the absence of molecular markers of iron limitation, indicated that the nitrogen depletion experiment was not iron limited. Metatranscriptomic samples (see SD5 and SD6 at the DOI mentioned above) were taken midbloom on day 3 Monterey Bay (MB1 and MB3) and late bloom on day 5 (MB2 and MB4) (Fig. 1).

While filtered counts of small cells showed an increase in haptophytes (mean values of 1.7 × 10^6^ cells L^−1^ midbloom to 3.1 × 10^6^ cells L^−1^ late bloom) and autotrophic flagellates (mean values of 1.6 × 10^6^ cells L^−1^ midbloom to 2.8 × 10^6^ cells L^−1^ late bloom), diatoms dominated large-cell counts (Fig. 2; see Fig. S2 at https://doi.org/10.5281/zenodo.6953574), total mRNA (Fig. 2), plastid 16S rRNA abundance (see Fig. S3 at the DOI mentioned above), and 18S rRNA abundance (see Fig. S4 and SD7 at the DOI mentioned above). An increase in fucoxanthin (39) from 1.35 μg/L to 58.91 μg/L confirms a diatom bloom (see Fig. S1 and SD2C at the DOI mentioned above). Pennate diatoms were the most abundant, increasing on average from 2.6 × 10^5^ cells L^−1^ midbloom to 1.6 × 10^6^ cells L^−1^ late bloom (see Fig. S2D at the DOI mentioned above). The pennate diatom community was dominated by toxin-producing Pseudo-nitzschia spp., which consistently expressed the domoic acid biosynthesis gene *dabA* (contig_503530_1_1053_+, contig_613449_1_1136_-; BLAST E value of <E−100) under all conditions, especially midbloom. The expression of *dabA* is frequently indicative of ongoing production of the toxin domoic acid (40–42). Centric diatoms also increased from 1.3 × 10^5^ cells L^−1^ to 9.6 × 10^5^ cells L^−1^ (Fig. 2) and were dominated by Chaetoceros and Thalassiosira spp. (see Fig. S2C at the DOI mentioned above). Despite cell counts depicting a diatom bloom, the proportion of centric diatom total mRNA did not significantly increase late bloom, and the proportion of pennate diatom total mRNA significantly decreased late bloom. This discrepancy could be due to the doubling of dinoflagellates, which have very large transcriptomes, and demonstrates the importance of collecting absolute count data when studying taxonomic composition.

Total mRNA (Fig. 2), 18S rRNA amplicons (see Fig. S8 at https://doi.org/10.5281/zenodo.6953574), and when applicable, plastid 16S rRNA amplicons (see Fig. S3 at the DOI mentioned above) showed that dinoflagellates, chlorophytes, cryptophytes, haptophytes, pelagophytes, fungi, and viruses were also highly active. Total mRNAs of Dinophyta, other alveolates, fungi, and viruses were significantly enriched (edgeR, false discovery rate [FDR] of <0.05) late bloom (MB2 and MB4) (Fig. 2; see SD8 at the DOI mentioned above). Dinoflagellates roughly doubled (from a mean of 2.7 × 10^3^ to 5.3 × 10^3^ cells L^−1^) and were dominated by the mixotrophic red tide species Akashiwo sanguinea (see Fig. S2B at the DOI mentioned above) (43). The cryptophyte-associated pigment alloxanthin increased over the bloom, as did the pelagophyte-associated pigment 19′-butanoyloxyfucoxanthin and the prymnesiophyte-associated pigment 19′-hexanoyloxyfucoxanthin (see Fig. S1 at the DOI mentioned above) (43). While prokaryotes were not visible in the poly(A)-enriched total mRNA data, cyanobacteria were present in 16S rRNA data (chiefly Synechococcus spp.) (see Fig. S5 at the DOI mentioned above), and the divinyl chlorophyll *a* concentrations indicated that Prochlorococcus also bloomed (see SD2 at the DOI mentioned above).

### Community response to iron depletion.

In the iron depletion experiment, the iron chelator deferoxamine B (DFB) induced iron limitation when added to a subsample of one of the early bloom incubations (barrel B4) (Fig. 1). As expected, total chlorophyll increased from 6.1 μg/L to 41.6 μg/L in the iron-replete barrel with a healthy variable fluorescence/maximum fluorescence (*F_v_*/*F_m_*) ratio of 0.5, whereas the iron-limited barrel did not bloom and the *F_v_*/*F_m_* ratio dropped to 0.24 (Fig. 1; see SD2B at https://doi.org/10.5281/zenodo.6953574). The growth observed in the iron-replete control barrel, along with the high nitrate concentrations (~30 μM) (Fig. 1B), indicates that the iron depletion experiment was not nitrogen limited at the time of molecular sampling. In the iron-replete barrel, diatom and dinoflagellate counts increased as in the nitrogen depletion experiment, although to a lesser extent (Fig. 2; see Fig. S2 at the DOI mentioned above). This may be because the surface water, which was the main component of incubations for the iron depletion experiment, had iron concentrations that were low and became limiting. In the iron-limited DFB-treated barrel, diatom cell numbers increased only modestly and were also dominated by *dabA*-expressing Pseudo-nitzschia spp. (see Fig. S2 at the DOI mentioned above). Dinoflagellate numbers were high in one Fe-replete replicate (MB7; 5.3 × 10^4^ cells L^−1^) and dropped by over 80% to 1.0 × 10^4^ cells L^−1^ under the Fe-depleted condition, implicating a mortality agent (see SD2D and Fig. S2B at the DOI mentioned above).

Dinoflagellate cell counts diminished under iron limitation (see Fig. S2B at https://doi.org/10.5281/zenodo.6953574), and they were the only group with a significant drop in relative mRNA abundance (edgeR FDR of <0.05) (Fig. 2). The relative mRNA abundance of chlorophytes also decreased under low iron (Fig. 2), although not significantly, perhaps because of iron stress. The concentrations of green alga photoprotective pigments zeaxanthin and lutein increased under iron limitation (see Fig. S1 at the DOI mentioned above). These pigments protect the LHC from oxidative damage during light stress (44) and have been shown to increase under iron limitation in land plants (45). Viral transcripts showed the reverse, significantly increasing with iron limitation (edgeR FDR of <0.05) (Fig. 2). In both experiments, the 16S rRNA community was dominated by common phycosphere lineages, including *Rhodobacteraceae*, *Flavobacteriaceae*, and a diversity of *Gammaproteobacteria* (46), and clustered by bloom state (see SD9 and Fig. S5 to S7 at the DOI mentioned above).

### Population-level selection.

While the genus-level mRNA composition of major algal lineages (see Fig. S8 at https://doi.org/10.5281/zenodo.6953574) did not change dramatically across either bloom, fine-scale taxonomic shifts were widespread. In order to examine population-level changes, we mapped reads to reference transcriptomes belonging to the 22 most abundant species annotations assigned to *ab initio* open reading frames (ORFs) (see Fig. S15 and SD10 and SD11 at the DOI mentioned above). For many reference species, a percent identity shift in mapped reads over the course of the bloom was conserved across replicates (see Fig. S9 and S10 at the DOI mentioned above). These conserved taxonomic shifts could indicate a rise in the dominance of specific subpopulations, but because the taxa observed here are distant from sequenced representatives, it is also possible that these shifts represent a regulatory change rather than a taxonomic one (e.g., upregulation of genes closer to known references during late bloom).

**FIG 3:**
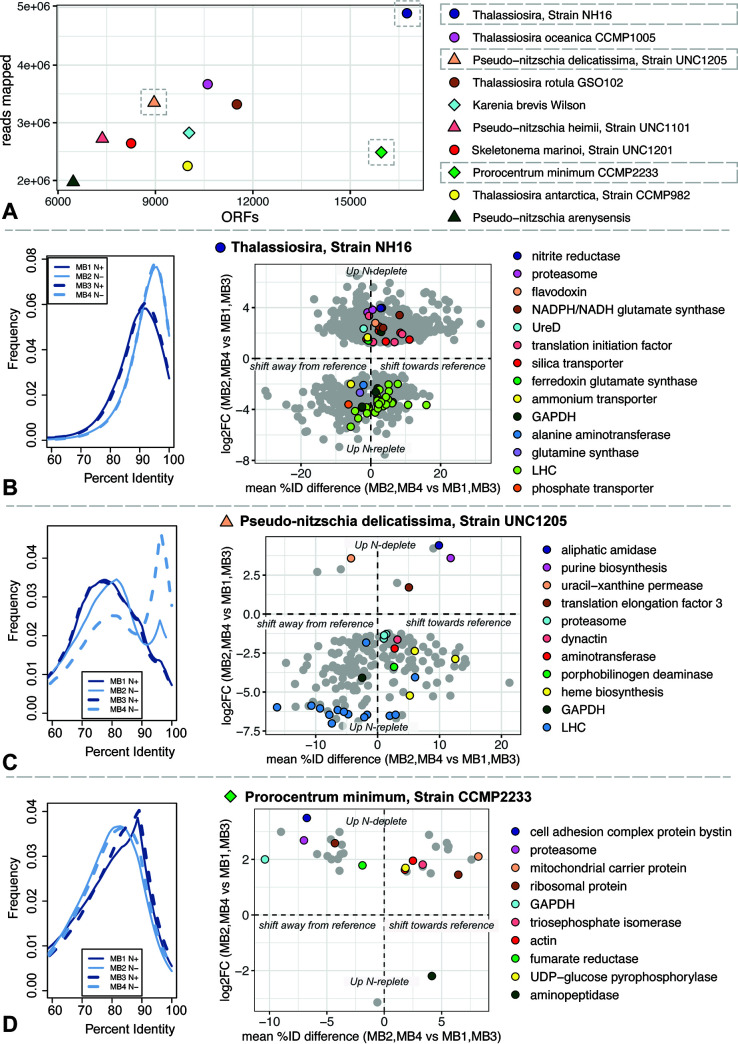
Fine-scale taxonomic shifts across the nitrogen depletion experiment. (A) Reads (*y* axis) mapping to and number of ORFs (*x* axis) mapped to top 10 most abundantly hit Marine Microbial Eukaryotic Transcriptome Sequencing Project (MMETSP) reference transcriptomes. Circles, triangles, and diamonds represent centric diatom, pennate diatom, and dinoflagellate references, respectively. (B to D) References are indicated with dashed gray lines. References are the centric diatom Thalassiosira strain NH16 (B), the pennate diatom Pseudo-nitzschia delicatissima strain UNC1205 (C), and the dinoflagellate Prorocentrum minimum strain CCMP2233 (D). Left, percent identity histograms of reads mapped to MMETSP reference contigs for all nitrogen conditions. Right, log fold changes (*y* axis) versus mean percent identity differences (*x* axis) for reads mapping to reference ORFs across nitrogen conditions. Only ORFs that are significantly differentially expressed, at least 60% identical to the reference, and shift in the same direction relative to the reference across replicates are shown. ORFs of interest are colored by function.

Population-level shifts were much more widespread in the nitrogen depletion experiment (see Fig. S9 at https://doi.org/10.5281/zenodo.6953574), where the bloom was more dramatic. To better understand which strain-level traits were adaptive, we examined ORF functions from the most abundant centric diatom, pennate diatom, and dinoflagellate references in the nitrogen depletion experiment. In particular, we examined ORFs that were significantly differentially expressed across bloom conditions and had conserved percent identity shifts across replicates (Fig. 3).

For both diatom references, reads were more similar to the reference under late bloom than under midbloom conditions in both replicates. Major functions driving this shift were light harvesting and carbon fixation (light-harvesting complex [LHC] genes, heme biosynthesis genes, porphobilinogen deaminase genes, and the glyceraldehyde-3-phosphate dehydrogenase gene *GAPDH*), as well as nitrogen acquisition and metabolism (ammonium transporters, glutamine synthases, and aminotransferases), which were significantly enriched midbloom. Diatoms are thought to outcompete other algae in bloom environments because of their high growth rate (47), proactive nitrogen uptake (19), and sophisticated nitrogen metabolism (21), so diatom subpopulations that upregulate light-harvesting and nitrogen metabolism genes more vigorously would have a competitive advantage.

The most abundant dinoflagellate reference was the common harmful algal bloom species, Prorocentrum minimum. A nanoscale secondary-ion mass spectrometry (nanoSIMS) experiment recently showed that this species had a large amount of population-level heterogeneity in nitrogen uptake, with various subpopulations displaying diverse modes of nutrition (48). Such physiological diversity in a starting population could explain the selective effect of the bloom. In contrast to the diatom references, there were few differentially expressed ORFs that could explain the percent identity shift away from the P. minimum reference. Dinoflagellates frequently respond to environmental changes at the protein level (49, 50), and many ORFs that appear to have undergone selection are involved in proteome remodeling. While some carbon fixation genes (*GAPDH* and triose-phosphate isomerase gene) were implicated in the percent identity shift, protein synthesis and degradation functions (aminopeptidases and proteasomes) and ribosomal proteins were more common.

### Gene-level selection.

In a bloom scenario, competition over fleeting nutrients presents an opportunity for selective sweeps in which the best-adapted clones dominate (37). To probe genic selection effects, single nucleotide variants (SNVs) were called on *ab initio* ORFs (see SD12 at https://doi.org/10.5281/zenodo.6953574) from both nitrogen and iron depletion experiments. SNVs were most abundant in centric diatoms and dinoflagellates, but other alveolates, pennate diatoms, and viruses had the highest percentages of nonsynonymous mutations (see Fig. S11A at the DOI mentioned above). In the nitrogen depletion experiment, there were replicated changes in the densities of SNVs (number of SNVs/total ORFs for a taxon group) over time. Diatom and chlorophyte SNV density decreased late bloom (see Fig. S11B at the DOI mentioned above), indicating that these groups underwent selection. In contrast, dinoflagellate SNV density increased late bloom, possibly because they did not bloom as dramatically as diatoms and therefore had less competition within their niche. Genetic recombination (37) probably also played a diversifying role. Dinoflagellate sexual reproduction marker *Sig* genes (51) were strongly upregulated midbloom. Algal sexual reproduction is often associated with nutrient limitation (52, 53), but it can be triggered by diverse stimuli (e.g., changes in light) (54, 55), and sexual reproduction under nutrient-replete conditions has been observed (56).

Dinoflagellate populations in both experiments had diverse protein regulatory machinery, with SNVs commonly occurring in ribosomal proteins, proteases, and histone-like proteins (annotated here as “bacterial DNA-binding proteins”; see Fig. S12 at https://doi.org/10.5281/zenodo.6953574) (57). Four variants of a centric diatom PP loop family Fe-S binding protein were more frequent in low iron (see Fig. S12, lavender, at the DOI mentioned above). Increased efficiency of these enzymes could confer a selective advantage in an iron-limited system. The greatest numbers of annotated nonsynonymous SNVs, though, were diatom and chlorophyte LHCs (see Fig. S12 at the DOI mentioned above). These SNVs were more frequent (abundant relative to other alleles at the same position) under replete conditions, implying that they were outcompeted by adaptive LHCs over the course of the bloom. Since regulation and modulation of light-harvesting machinery is a critical factor in algal nitrogen and iron responses, it is not surprising that genes with these functions underwent the greatest selection.

SNVs of a viral RNA-dependent RNA polymerase (*RdRp*; contig_1975898_1_2441_+) (see Fig. S12A at https://doi.org/10.5281/zenodo.6953574) that forms a clade with brown alga viruses (see Fig. S13 at the DOI mentioned above) rose to dominance late bloom in both experiments. These fixed *RdRp* alleles are likely associated with successful viral phenotypes and could be infecting diatoms, which dominated the bloom.

### Response to nutrient stress varied taxonomically.

**FIG 4:**
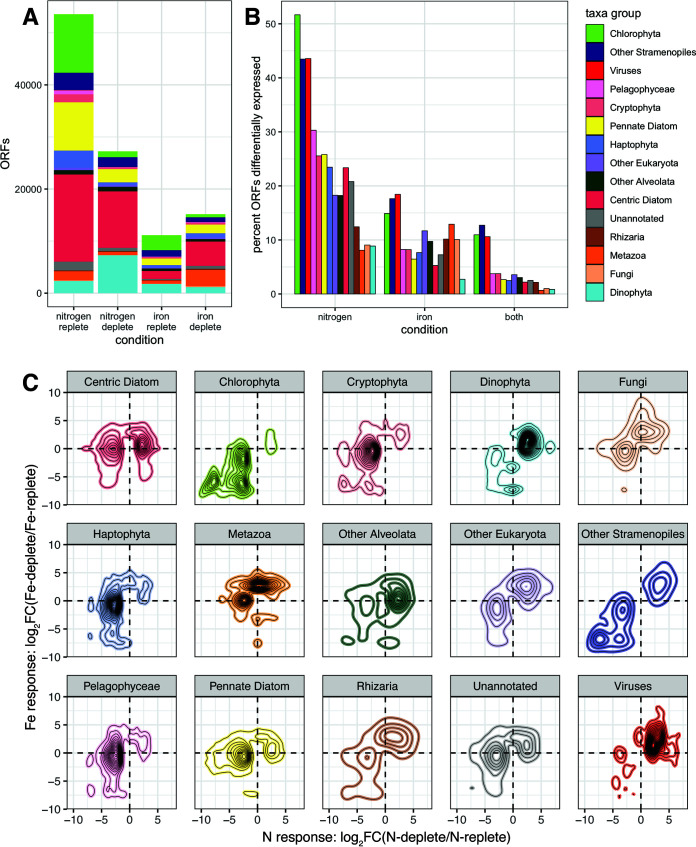
(A) Numbers of ORFs significantly upregulated under nitrogen-replete, nitrogen-depleted, iron-replete, and iron-depleted conditions, colored by taxonomy. (B) Percentage of ORFs from each taxon group that are significantly differentially expressed across nitrogen conditions, iron conditions, and both types of nutrient limitation. (C) Two-dimensional histograms characterizing the transcriptional responses of ORFs from major lineages to nitrogen and iron status (*y* axis, log_2_FC(Fe depleted/Fe replete), >0 is up in low iron, <0 is down in low iron) and nitrogen (*x* axis, log_2_FC(N depleted/N replete), >0 is up in low nitrogen, <0 is down in low nitrogen). Only ORFs that were significantly differentially expressed (FDR < 0.05) under at least one condition (N, Fe, or both) are included.

Nearly a quarter of ORFs were significantly nutrient responsive (see SD5 at https://doi.org/10.5281/zenodo.6953574). Of these, most were differentially expressed (DE) in response to nitrogen limitation (84%), with fewer (27%) responsive to iron (see SD5 at the DOI mentioned above). Sixty-six percent of nitrogen-responsive ORFs were downregulated at low N (*n* = 53,549) (Fig. 4A), consistent with decreased energetic, metabolic, and growth processes during nitrogen limitation. In contrast, iron-responsive ORFs were more often upregulated at low Fe (*n* = 15,171, 58% of ORFs differentially expressed in response to Fe), and many were involved with iron scavenging.

The intensity of the transcriptional response to nutrient limitation varied taxonomically (Fig. 4B). Dinoflagellates were the least responsive taxon group (14.3% of DE ORFs), consistent with the view that they respond to environmental perturbations primarily posttranscriptionally (49, 50). Both centric and pennate diatoms had a high density of both N- and Fe-responsive ORFs across a 5-log2 fold change (log_2_FC) range under both conditions (Fig. 4C). Many ORFs from dinoflagellates and viruses, which prosper during the demise of a diatom bloom (58–61), increased in expression late bloom.

### Light-harvesting machinery was remodeled under nutrient stress.

**FIG 5:**
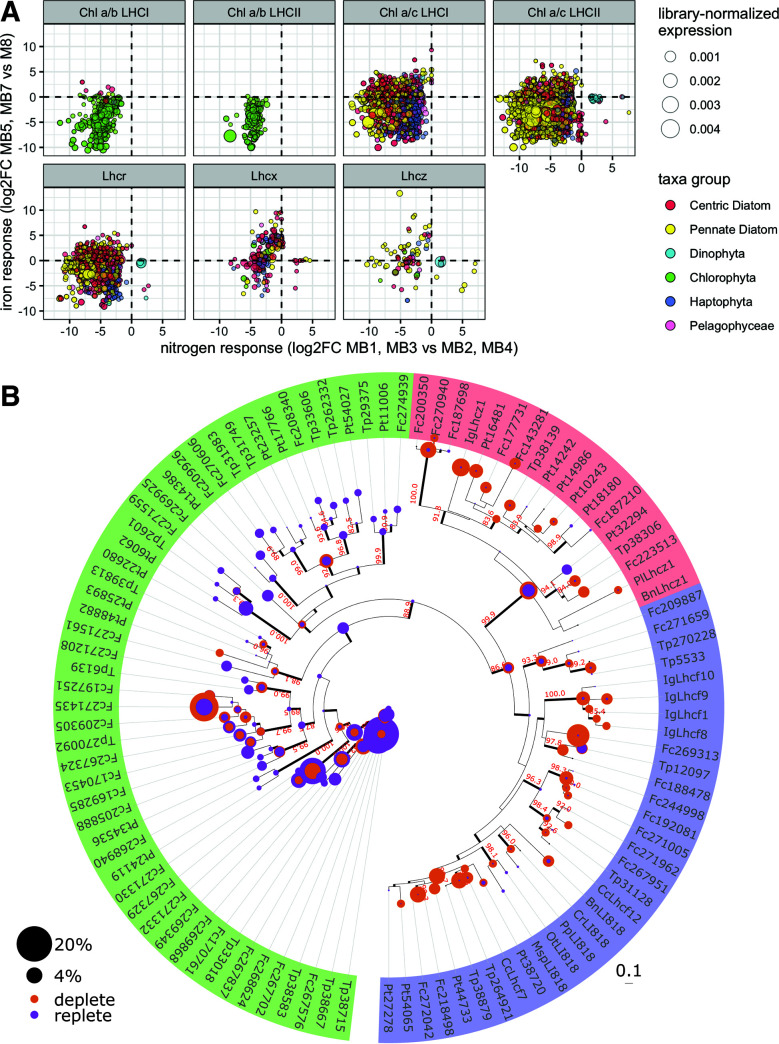
Responsiveness of light-harvesting complex (LHC) to nutrient stress. (A) Responses of LHC subfamilies to iron (*y* axis; >0 is up in low iron, <0 is down in low iron) and nitrogen (*x* axis; >0 is up in low nitrogen, <0 is down in low nitrogen). Chlorophyll is abbreviated “Chl.” Only LHC ORFs that are significantly differentially expressed (FDR < 0.05) under at least one condition are shown. (B) Phylogenetic tree showing expression of LHC transcripts in nutrient-replete conditions (MB1, MB3, and MB7; purple circles) versus nutrient-depleted conditions (MB2, MB4, and MB8; orange circles). The outer ring of the phylogenetic tree is colored according to LHC family (green is non-stress responsive, red is Lhcz, and blue is Lhcx [also known as Lhcsr or LI818]).

LHCs were the most obvious responders to nutrient status. Our custom LHC hidden Markov model (HMM) identified 14,835 taxonomically diverse LHC ORFs, of which 7,882 were significantly nutrient responsive (53%; chi-square test, *P* < 2.2e−16): 97.6% of nutrient-responsive LHCs were significantly downregulated in low nitrogen (*n* = 7,622) or iron (*n* = 747) (Fig. 5; see SD13 at https://doi.org/10.5281/zenodo.6953574). This is consistent with overall reduction of photosynthesis in response to a shortage of either macromolecules that are essential for growth (62) or iron, which is essential for photosynthetic electron transport (63). Chlorophyte LHCs were more commonly downregulated in low iron (36.47% of LHCs) than dinoflagellate, pelagophyte, haptophyte, and diatom LHCs (0.65 to 9.19%) (Fig. 5A) (64), suggesting that either red alga lineages are less impacted by iron limitation or chlorophytes strategically downregulate light harvesting at low iron.

LHCs play an important role in photoprotection, dissipating excess energy via nonphotochemical quenching (NPQ). In Chlamydomonas reinhardtii (65) and diatoms (66), the stress responsive Lhcsr/Lhcx family (here, Lhcsx) has been shown to be essential for this process. Contrary to the general trend, the photoprotective *Lhcx* (34, 67–69) and sister *Lhcz* clade (70) were strongly upregulated in low Fe (maximum log_2_FC of ~10) (Fig. 5), suggesting an overall shift in the quality of the LHC from a photosynthetic to a photoprotective configuration, leading cells toward an energy-dissipative state at low Fe (71, 72). *Lhcx* ORFs significantly enriched in low Fe pertained to diatoms (*n* = 112), haptophytes (*n* = 30), pelagophytes (*n* = 10), chlorophytes (*n* = 2), and dinoflagellates (*n* = 2) (see SD13 at https://doi.org/10.5281/zenodo.6953574), indicating that this mechanism may be more broadly utilized than previously known. The Lhcz family has not been explicitly implicated in photoprotection before. However, *Lhcz*’s significant upregulation at low iron suggests that it is an important photoprotective mechanism in diatoms (*n* = 26 ORFs) and haptophytes (*n* = 8 ORFs) (see SD9 at the DOI mentioned above).

The conformational change of LHCs during NPQ is triggered by the binding of embedded xanthophyll pigments, which are de-epoxidated as pH drops when photosynthetic electron transport exceeds CO_2_ fixation capacity (73). Since transitions between epoxidated and de-epoxidated xanthophyll pigments are enzymatically catalyzed, we searched for putative xanthophyll epoxidase and de-epoxidase transcripts. These transcripts were significantly downregulated at low nitrogen in chlorophytes, diatoms, and other stramenopiles, suggesting that the ability to cycle xanthophylls is reduced alongside light harvesting (see SD5 at https://doi.org/10.5281/zenodo.6953574). Dinoflagellates and haptophytes had largely unresponsive xanthophyll cycle enzymes. Only diatoms significantly upregulated xanthophyll cycle transcripts in low iron, which may confer an advantage in iron-limited regimes. This is consistent with iron-limited cultures of Thalassiosira oceanica, which significantly upregulated xanthophyll cycle de-epoxidase proteins (69).

### Metabolism and nutrient uptake were sensitive to bloom state.

**FIG 6:**
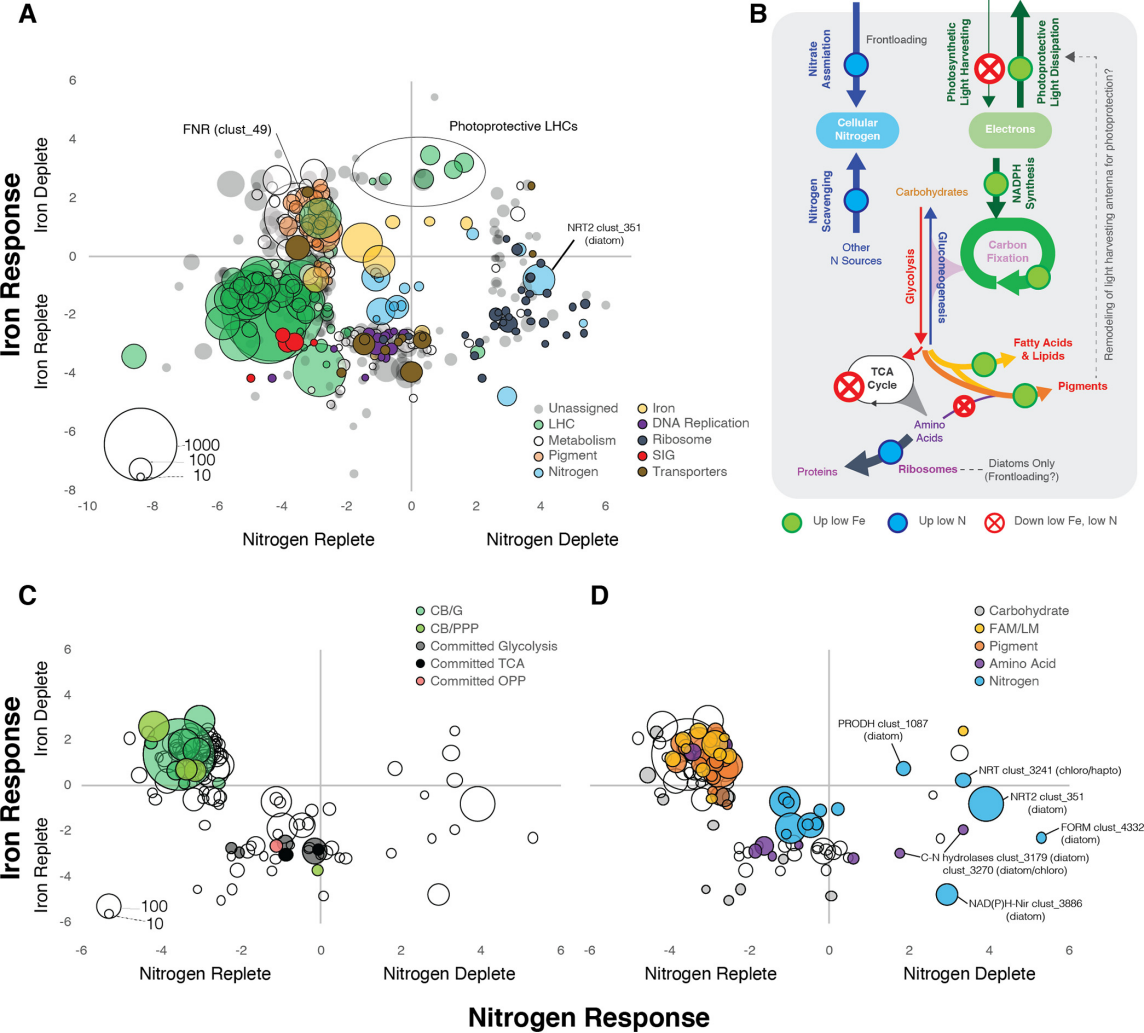
(A) Responses of major clusters of diatom, dinoflagellate, chlorophyte, haptophyte, and pelagophyte ORFs to iron and nitrogen status. The average log_2_FC of ORFs within each cluster is shown across nitrogen conditions (*x* axis) and iron conditions (*y* axis). Clusters were filtered by the following criteria: >10 ORFs, at least one significantly differentially expressed ORF for either N or Fe per cluster (taxon group-wise edgeR FDR of <0.05), and average cluster abs(log_2_FC) of >2.5 for either N or Fe. Clusters were assigned to general cellular functions based on annotations of ORFs within the clusters (see SD15 at https://doi.org/10.5281/zenodo.6953574). Some clusters with functions in iron and nitrogen metabolism that did not meet the abs(DE) of >2.5 criteria are also plotted. LHC, light-harvesting complex; SIG, sexually induced gene. (B) Integrated model of energetic inputs and pathway flux (inferred from transcripts). (C, D) Open circles show metabolism gene clusters (from panel A), colored according to assignment to different pathways. abs, absolute value; CB, Calvin Benson; G, glycolysis/gluconeogenesis; PPP, pentose phosphate pathway (oxidative or reductive); TCA, tricarboxylic acid cycle; OPP, oxidative pentose phosphate; FAM/LM, fatty acid metabolism/lipid metabolism.

Only 6.9% of ORFs (*n* = 27,219) were significantly upregulated at low nitrogen. Chief among these were ORFs involved in nitrogen uptake and assimilation (e.g., the nitrate transporter gene *NRT2* and nitrate reductase and nitrite reductase genes) (Fig. 6; see Fig. S14 and S15 at https://doi.org/10.5281/zenodo.6953574). Formamidase ORFs were also strongly and consistently upregulated at low N across lineages. The upregulation of formamidase in low N has been observed in the laboratory and field in diverse phytoplankton (16, 33, 74–76) (see Fig. S14 and S15 and SD14 at the DOI mentioned above). Formamidase produces formate and ammonium from formamides, which are produced during histidine and cyanide catabolism (77). The specific function of formamidase in phytoplankton biology is unknown; however, its upregulation under N-depleted conditions points to a role in recovering nitrogen from organic sources (intracellular or extracellular) when inorganic sources are unavailable.

In the iron limitation experiment, the *F_v_*/*F_m_* ratio indicated that limitation was stressful enough to impact photosystem function. While the *F_v_*/*F_m_* ratio was relatively stable at 0.5 under iron-replete conditions (B2, nutrient amended and unchelated), the addition of an iron chelator to B4 (100 nM DFB) reduced the *F_v_*/*F_m_* ratio to 0.24 (see Fig. S2 at https://doi.org/10.5281/zenodo.6953574). Iron starvation-induced protein genes (*ISIP*s) were expressed in diatoms and other taxa, including the carbonate-dependent inorganic iron transporter gene *ISIP2A* (encoding a protein also known as phytotransferrin) (78), consistent with previous observations (see Fig. S16 and S17 and SD14 at the DOI mentioned above) (34–36). However, since inorganic iron is insoluble in seawater, most bioavailable iron in the water column is complexed by organic ligands (79). Transcripts for ferric reductases, which reduce and import this complexed extracellular iron (80), were detected in diatoms, haptophytes, chlorophytes, and dinoflagellates. Only diatoms and haptophytes significantly upregulated ferric reductase ORFs at low iron (2.3 < log_2_FC < 9.3), possibly indicating the possession of a more sensitive iron-sensing system in these groups (81, 82). Replacements for iron metalloenzymes, such as plastocyanin, the copper-containing alternative to cytochrome *c*_6_ (83), and flavodoxin, the flavin-containing alternative to ferredoxin (84), were also expressed in low iron (see SD5 at the DOI mentioned above). Conversely, when iron was replete, increased light-harvesting capacity was accompanied by increased carbon fixation and sugar storage (e.g., upregulation of *PEPC* and the transketolase gene) and upregulation of genes encoding iron-dependent enzymes (e.g., Fe-Mn SOD and biotin synthase genes), consistent with other studies (see Fig. S31 and S32 at the DOI mentioned above) (34–36).

To compare the physiological nutrient response of major lineages while accounting for changes in abundance, differential expression analysis was run groupwise on 161,044 diatom, dinoflagellate, chlorophyte, haptophyte, and pelagophyte ORFs (Fig. 6). These ORFs were then assigned to ortholog clusters using orthoMCL (*n* = 48,114) (see SD15 and SD16 at https://doi.org/10.5281/zenodo.6953574). Cluster DE values were calculated (average of all within-cluster ORFs) for clusters with >10 ORFs (*n* = 5,664 clusters), which were manually classified into broad functional categories (see SD16 at the DOI mentioned above). Major responses, e.g., downregulation of *LHC* and *Sig* genes in limiting iron and nitrogen, were consistent with observations at the ORF level, but the cluster-level approach highlighted additional functional changes.

Several clusters involved in DNA replication (DNA polymerase, DNA primase, and MutS mismatch repair protein genes) (see SD16 at https://doi.org/10.5281/zenodo.6953574) were strongly downregulated during iron limitation and less strongly by nitrogen limitation, identifying part of the molecular mechanism used to slow growth under unfavorable conditions (Fig. 6A; see SD16 at the DOI mentioned above). Another notable feature was the upregulation of clusters involved in diatom ribosome biogenesis and assembly (i.e., pescadillo, bystin, and BRX1 genes) in low N, along with the diatom *NRT2*, other key nitrate assimilation genes, formamidase genes (also observed at the ORF level), and proline dehydrogenase gene *PRODH* (Fig. 6D). Upregulation of nitrate assimilation genes has been observed in natural diatom communities during limiting nitrogen and is thought to allow for both frontloading transcripts in preparation for new nitrogen inputs and scavenging nitrogen (85). Upregulation of ribosomes at low N may either be the diatom response to enhanced intracellular recycling and remodeling of the proteome or an attempt to be competitively poised for the next influx of new nitrogen, as they do with *NRT2* and other nitrate assimilation genes (85).

Only one component of the photosynthetic electron transport (PET) chain, ferredoxin-NADP(+) reductase (*FNR*, cluster 49) was upregulated at low Fe (Fig. 6A). FNR catalyzes the final step of PET to transfer an electron from reduced ferredoxin to generate the NADPH used to drive carbon fixation. Upregulation of *FNR* at low Fe may represent a strategy used across different phytoplankton taxa to try to sustain high levels of NADPH production in response to reduced availability of ferredoxin and reduced PET (Fig. 6). Taken together, the reduced LHC, enhanced pigment and Lhcr, and increased FNR point to molecular remodeling of the photosynthetic apparatus across diverse taxa to tune energy dissipation and production to overall nutrient status.

Nutrient-induced shifts in metabolism are also evident at the cluster level (Fig. 6C and D). Several clusters comprised of key committed glycolysis and tricarboxylic acid (TCA) cycle enzymes were downregulated under nutrient limitation, consistent with a reduction of overall metabolism and growth (Fig. 6B and C). Most clusters annotated with putative roles in the Calvin-Benson cycle (e.g., glyceraldehyde phosphate dehydrogenase [*GAPDH*] and phosphoribulokinase [*PRK*]) and fatty acid biosynthesis were downregulated at low N but upregulated at low Fe, similar to *FNR* and pigment biosynthesis (Fig. 6), likely representing an effort to sustain flux through these pathways despite the reduction of energy inputs [ATP and NAD(P)H] imposed by acute iron limitation. Furthermore, these carbon pools represent cellular stores for ATP and reductant and upregulating their expression may be a strategy to stockpile chemical energy in anticipation of surviving more severe or longer-term Fe limitation.

### Genetic markers of nitrogen and iron status identified in diatoms.

Oceanographic investigations of phytoplankton physiology would benefit from the existence of robust and incubation-independent biomarkers of *in situ* physiology, yet very few of these biomarkers have been reliably identified. Some recommendations have been made for field gene markers to detect the cellular iron status (e.g., flavodoxin and iron starvation-induced protein genes). Iron starvation-induced proteins (ISIP1, ISIP2/phytotransferrin, and ISIP3) (78, 86) and the metalloprotein replacement, flavodoxin (84) have been shown to be highly responsive to iron starvation in diatoms in the laboratory and in the field (32, 34, 36, 87). Iron-limited diatoms also have a reduced capacity for nitrogen assimilation because many nitrogen assimilation enzymes require iron (35, 36). There are not yet reliable indicators of cellular nitrogen status, however (88, 89). Key nitrogen uptake and metabolism genes are transcribed during both nitrogen starvation and availability, rendering them ineffective as a diagnostic tool on their own (16, 17, 90). Additionally, nitrogen limitation strongly impacts global gene expression, commonly inducing significant DE over half of the genome, further complicating the identification of specific nitrogen status biomarkers (16, 17, 74). Here, we identify pairs of genes with consistently opposing responses to nitrogen and iron limitation, the ratios of which can be used to indicate the nutrient status of the cell.

Recent work in the model diatom Phaeodactylum tricornutum has shown that, while most nitrate assimilation genes (i.e., *NRT2* and *NR*) are expressed in response to both nitrate and nitrogen deprivation, the glutamine synthase gene (*GSII*; assimilates ammonium into glutamine) is upregulated specifically in the presence of nitrate and not in response to other N sources or nitrogen deprivation (16, 17). Therefore, using the ratio of *NRT2* or *NR* to *GSII* transcripts should serve as an indicator of intracellular nitrogen status. Indeed, we observed a strong upregulation of *NRT2* (cluster 351) and downregulation of *GSII* (cluster 139) under N-depleted conditions, consistent with patterns seen in nitrogen-limited cultures of P. tricornutum (see Fig. S18A at https://doi.org/10.5281/zenodo.6953574). This index not only indicates nitrogen status at the community level (clusters) but can also be calculated from ORFs assigned at the genus level, meaning it has the potential to resolve differences in the nitrogen status of populations within the diatom community (see Fig. S19A at the DOI mentioned above). Additionally, this pattern held at the taxon group level for all groups except dinoflagellates and may be a useful tool for phytoplankton more generally (see Fig. S18B at the DOI mentioned above).

For iron, we surveyed the expression of genes that were historically found to be iron responsive and genes that require iron as cofactors. We found that the ratio of total *ISIP* expression (*ISIP1*, UniProt B7GA90; *ISIP2*, UniProt B7FYL2; and *ISIP3*, UniProt B7G4H8) to the expression of the thiamine (vitamin B_1_) synthesis ORF, phosphomethylpyrimidine synthase (*thiC*; K03147) was consistently higher for Fe-depleted than for Fe-replete conditions across diatom groups (see Fig. S19B at https://doi.org/10.5281/zenodo.6953574). In natural diatom populations, *ISIP*s were upregulated in response to experimentally iron-depleted water, and *thiC* was upregulated in response to added iron (35, 36). We found this ratio to more consistently identify iron status than either the ratio of flavodoxin to ferredoxin genes (84) or the ratio of *ISIP2* to the ferritin gene (88). Additionally, the expression of both *ISIP*s and *thiC* was downregulated in response to low N to a similar degree, meaning that the ratio is unlikely to be affected by nitrogen status. While the ratio of *ISIP*s to *thiC* performs well for diatoms, this metric may not be as informative for other taxa that rely on the acquisition of environmental thiamine rather than producing it endogenously.

### Viral infection as a driver of bloom demise.

**FIG 7:**
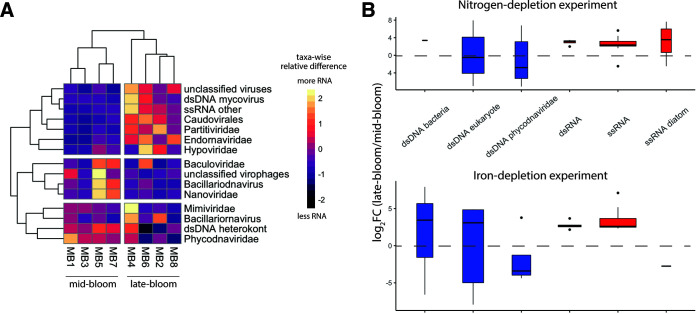
(A) Heatmap of most abundant viral taxa across experimental conditions. (B) Log_2_FC in expression under late-bloom conditions relative to early-bloom conditions for both experiments. Values for DNA viruses are shown in blue, and values for RNA viruses are shown in red.

Viruses with putative hosts that included bacteria, fungi, heterotrophic flagellates, and diverse algae were detected in both experiments (see Fig. S20 at https://doi.org/10.5281/zenodo.6953574) and may have been an even larger part of the community than we observed, due to the tendency of polyadenylation-selected libraries to produce fewer viral contigs (91). Regardless of whether the bloom was being limited by nitrogen or iron, overall viral RNA was higher late bloom than midbloom (Fig. 2). This could be because nutrient-stressed cells were more prone to infection, rapid replication of host cells allowed viruses to flourish, or high cell density allowed increased contagion. It could also be because diverse RNA viruses dominated viral expression (Fig. 7B). RNA virus RNA represents not only expression, but also genomic material. Therefore, RNA viruses may show a stronger signal later in the bloom because of an accumulation of biomass and not necessarily greater activity.

While viral RNA was up at late bloom for nearly all lineages, RNA corresponding to double-stranded DNA (dsDNA) viruses infecting bacteria and eukaryotes was up midbloom in some cases, including for the Phycodnaviridae lineage infecting algae (Fig. 7A). Bacteriophages and dsDNA mycovirus-annotated RNA were also up late bloom, perhaps increasing alongside heterotrophic bacteria and fungi feeding on lysed cells.

While dinoflagellate viruses were not specifically identified, diatom viruses were. For example, there was a dramatic increase in RNA best annotated as a single-stranded RNA (ssRNA) diatom virus at late bloom in the nitrogen depletion experiment (Fig. 7B). This indicates that viral infection, along with nutrient limitation, likely played a role in bloom demise.

### Conclusions.

Because diatoms often dominate during coastal upwelling, they have the best characterized transcriptional response to nitrogen and iron (5, 16–18, 21, 34–36, 63, 68, 78, 80, 89). Here, while diatoms were the most obvious blooming taxa in terms of settled cell counts, filtered counts of small cells revealed that haptophytes and autotrophic flagellates also bloomed, and mRNA confirmed that dinoflagellates, chlorophytes, cryptophytes, haptophytes, pelagophytes, fungi, and viruses were also highly active. We find that, while each lineage has a unique pattern of transcriptional response to nitrogen and iron (Fig. 4), some responses are similar across groups, suggesting that many strategies used by diatoms to cope with low N (e.g., assimilating and scavenging nitrogen and downregulating light harvesting and Calvin-Benson cycle transcripts) and low Fe (e.g., remodeling the light harvesting complex for photoprotection, sustaining NADPH production and flux through the Calvin-Benson cycle, and upregulating iron acquisition genes and alternate metalloenzymes) are also employed by diverse phytoplankton taxa.

However, some nutrient response strategies were unique to diatoms. For example, diatoms upregulated ribosome biogenesis and assembly transcripts in response to low N, possibly to enhance intracellular recycling and remodeling of the proteome or to be competitively poised for the next influx of new nitrogen (85). Additionally, only diatoms had significantly enriched xanthophyll cycle transcripts in response to low Fe.

While the effect of nutrient limitation on phytoplankton cells has been studied at the transcript level, the selective pressure that nutrient limitation exerts on phytoplankton at the population level has largely been overlooked (37, 38). Here, we observed replicated population-level shifts as the blooms progressed. Shifted ORFs appeared functionally adaptive to bloom conditions, likely due to both increased abundance of winning taxa and overexpression of adaptive genes within already abundant species. Diatoms and chlorophytes appeared to have undergone selection in the nitrogen depletion experiment (decreased SNV density), whereas dinoflagellates, which showed the strongest upregulation of sexual reproduction genes, appeared to have undergone diversification (increased SNV density).

Infection status is not often considered when studying phytoplankton nutrient response (32, 35, 36, 89), but here we identified RNA mapping to viruses of diverse phytoplankton lineages, including diatoms, dinoflagellates, haptophytes, and chlorophytes. Viral RNA was proportionally more abundant later in both blooms and likely exacerbated nutrient stress and contributed to bloom demise.

Nitrogen and iron are the most widespread nutrients limiting marine phytoplankton growth. Nutrient stress can be difficult to monitor in the field (88), so here we suggest the ratio of total *ISIP*s to *thiC* as a biomarker of iron stress in diatoms and present for the first time a putative biomarker of diatom nitrogen status: the ratio of *NRT2* to *GSII*. Together, we present a snapshot of not only the physiological response of phytoplankton to nutrient conditions but, also, the dynamic genetic selection processes by which these responses were established.

## MATERIALS AND METHODS

Two incubation experiments (Fig. 1) were conducted to measure the impact of nitrogen and iron drawdown on phytoplankton succession and physiology. Previously described bloom simulation methods (47, 92) were implemented with seawater from Monterey Bay, California (36°50.72′N, 121°57.89′W), in September 2008. Seawater was incubated for 6 days under ambient light and temperature. Pigments, pH, nitrate, and nitrite were monitored throughout both experiments as previously described (see SD1 and SD2 at https://doi.org/10.5281/zenodo.6953574) (92).

The nitrogen depletion experiment simulated upwelling in duplicate (barrels 1 and 3) by combining 1 L of 6-m surface water and 199 L of 70-m subnutricline water (Fig. 1A, red and blue). Cell counts (as previously described [92]) and molecular samples were collected midbloom (nitrogen replete, day 3) and late bloom (nitrogen depleted, day 5).

The iron depletion experiment amended 200 L of surface water with 40 μM nitrate, 2.5 μM phosphate, and 50 μM silicon (final concentrations) in barrel 2 (Fig. 1A; green). Cell counts and molecular samples were collected midbloom (day 2), after which 20 L was subsampled and incubated in barrel 4 (Fig. 1A, purple) with the iron chelator deferoxamine B (DFB; binding constant, 10^30^) at a final concentration of 100 nM (93). In barrel 2, the iron levels were left unmanipulated as a control. Both barrels were sampled again at late bloom (day 4).

Full methods are in Text S1 in the supplemental material. Briefly, 2 L of water per sample was filtered through a 0.22-μm filter for a TRIzol (Life Technologies; Carlsbad, CA) RNA extraction. Amplicon libraries were constructed from cDNA generated with the SuperScript III first-strand cDNA synthesis system using 341F and 926R primers for 16S rRNA and TAReuk454FWD1 and TAReukREV3 primers for 18S rRNA. Poly(A) mRNA metatranscriptomic libraries were constructed with 0.8 μg of total community RNA using the TruSeq RNA kit version 2 (Illumina) and sequenced using the Illumina HiSeq.

10.1128/msystems.00729-22.1 TEXT S1 Supplementary materials and methods. Download Text S1, PDF file, 0.1 MB. Copyright © 2022 Kolody et al. 2022 Kolody et al. https://creativecommons.org/licenses/by/4.0/ This content is distributed under the terms of the Creative Commons Attribution 4.0 International license.

### Bioinformatics.

Reads were processed via the RNAseq Annotation Pipeline (RAP) (94) as previously described (95) (see SD3 at https://doi.org/10.5281/zenodo.6953574). After trimming and quality filtration, rRNA was removed with riboPicker version 0.4.3 (96) and reads were assembled, first by library and then overall, with CLC Genomics Workbench 9.5.3 (https://www.qiagenbioinformatics.com/). *Ab initio* ORFs were predicted with FragGeneScan and then screened for rRNA, ITS, primers, and organellar ORFs. Light-harvesting complex (LHC) sequences were identified specifically using custom HMMs (https://github.com/allenlab/Data_Files/blob/main/LHC_hmm). Reads were also mapped directly to reference transcriptomes that commonly appeared as best hits for *ab initio* ORFs with the Burrows-Wheeler Aligner algorithm BWA-MEM (97, 98). ORFs from these references were clustered with *ab initio* ORFs from the five major phytoplankton groups (centric diatoms, pennate diatoms, chlorophytes, haptophytes, and pelagophytes) to form peptide ortholog groups via the Markov Cluster Algorithm (MCL; https://micans.org/mcl/) (99).

In order to statistically distinguish genes expressed more highly in replete versus depleted conditions, differential expression (DE) was assessed using edgeR version 3.16.5 (100). Counts were normalized using trimmed mean of M values (TMM), and pairwise comparisons were made using the quantile-adjusted conditional-likelihood (qCML) method for estimating tagwise dispersions and performing an exact test for the negative binomial distribution (100). For the nitrogen depletion experiment, N-depleted samples MB2 and MB4 were compared to N-replete controls MB1 and MB3. For the iron depletion experiment, Fe-depleted sample MB8 was compared to Fe-replete controls MB5 and MB7. These results were validated by comparing our mostly highly DE genes to those found to be differentially expressed across nitrogen and iron conditions in existing phytoplankton transcriptomes (16, 33–36, 74, 101) and by checking the behavior of genes typically responsive to nitrogen (e.g., formamidase and urease genes, *TKL*, *FBA*, *FBP*, *PRK*, *TPI*, *RPE*, *PGK*, and *PETH*) and iron (e.g., *ISIP*s and ferredoxin and flavodoxin genes).

### Data availability.

Single nucleotide variants (SNVs) were detected among reads mapping to *ab initio* ORFs using samtools mpileup (102). Raw data are at NCBI BioProject accession number PRJNA715489 under BioSample accession numbers SAMN18381652 to SAMN18381659 (18S rRNA), SAMN18381676 to SAMN18381683 (16S rRNA), and SAMN18381589 to SAMN18381596 (metatranscriptomes). Curated data (data sets SD1 to SD16) and supplemental figures are available at https://doi.org/10.5281/zenodo.6953574.

10.1128/msystems.00729-22.2 TABLE S1 Abbreviations used in the text. Download Table S1, PDF file, 0.03 MB. Copyright © 2022 Kolody et al. 2022 Kolody et al. https://creativecommons.org/licenses/by/4.0/ This content is distributed under the terms of the Creative Commons Attribution 4.0 International license.
