# Supplementary material for: Nitrogen and Iron Availability Drive Metabolic Remodeling and Natural Selection of Diverse Phytoplankton during Experimental Upwelling
Source: mSystems. 2022 Aug 29;7(5):e00729-22. doi: 10.1128/msystems.00729-22 (PMC9599627; doi:10.1128/msystems.00729-22)
Supplement: TEXT S1 [file msystems.00729-22-s0001.pdf]

Supplementary File 1: Supplementary Text

Supplementary Materials and Methods

Light Microscopy

Phytoplankton species were identified and counted by light microscopy in samples preserved in 1% paraformaldehyde. Cell counts were normalized to cells/L.
Large cells such as diatoms, dinoflagellates, and ciliates were separated using the Utermöhl settling technique. Smaller cells were collected onto a 0.8 um filter (1).

Metatranscriptome library preparation and sequencing

For molecular analyses, approximately 2 L of water for each sample was filtered onto 0.22-µm sterivex filters. Filters were frozen in liquid nitrogen, kept on dry ice for shipping and stored in the laboratory at -80ºC. RNA was purified from filters using the Trizol reagent (Life Technologies; Carlsbad, CA), and treated with DNase (Qiagen, Valencia, CA, USA) and cleaned with the RNeasy MinElute Kit (Qiagen, Valencia, CA, USA). RNA quality was analyzed with on a 2100 Bioanalyzer with Agilent RNA 6000 Nano Kits (Agilent Technologies, Santa Clara, CA, USA) and quantified using Qubit Fluorometric Quantification system (TheroFisher, Waltham, MA, USA).

After setting aside 1 µg total RNA from each sample for ribosomal RNA amplicon sequencing, PolyA mRNA transcriptomes were constructed with 0.8 µg of total community RNA using TruSeq RNA Library Preparation Kit v2 (Illumina™), following the manufacture's protocol with minor adjustments. Specifically, fragmentation time was modified according to RNA quality. Library quality was analyzed on a 2100 Bioanalyzer with Agilent High Sensitivity DNA Kits (Agilent Technologies, Santa Clara, CA, USA).

The mean size of the libraries was around 400 base pairs. Resulting libraries were subjected to paired-end sequencing via Illumina HiSeq.

Amplicon library preparation and sequencing

1 µg total RNA from each sample was converted to cDNA using the SuperScript-III First Strand cDNA Synthesis System with 1 µL random hexamer primers. 16S and 18S rRNA PCR amplifications were each performed using the Life Technologies AccuPrime PCR system kit in reactions containing 1 µL of cDNA per sample as a template, 1X AccuPrime Buffer I, 0.15 µL AccuPrime Taq High Fidelity, and a final primer concentration of 200 nM. A no-template negative control for cDNA synthesis was also included. To amplify 16S rRNA, nearly-universal bacterial primers 341F (5'-CCTACGGGNGGCWGCAG-3') (2) and 926R (5'-CCGTCAATTCMTTTRAGT-3') (3) were used to target an approximately 500 bp segment the v3v5 region. To amplify 18S rRNA, TAReuk454FWD1 (5′-CCAGCASCYGCGGTAATTCC-3′) and TAReukREV3 (5′-ACTTTCGTTCTTGATYRA-3′) (4) primers were used to target an approximately 500 bp segment of the v4 region. Both primer sets were adapted for multiplexed sequencing with the addition of FLX Titanium adapters (A adapter sequence: 5′ 127 CCATCTCATCCCTGCGTGTCTCCGACTCAG 3′; B adapter sequence: 5′ 128 CCTATCCCCTGTGTGCCTTGGCAGTCTCAG 3′) and 10bp multiplex identifier (MID) barcodes. PCR cycling conditions consisted of an initial denaturation at 95°C for 2 minutes, 30 cycles of 95°C for 20 seconds, 56°C for 30 seconds, and 72°C for 5 minutes. PCR products (3 µl of each sample and 5 µl of negative control) were run on a 1% agarose gel at 110 V for 70 minutes, cleaned up using the AMPure XP bead kit (Beckman Coulter Life Sciences, Brea CA), and resuspended in 25 µL of Qiagen elution buffer (EB). The final product was visualized for quality assessment on an agarose gel (2.5 µL) and quantified using in a LifeTechnologies'

PicoGreen Quant-IT assay (1 µL). Using this quantification, 20 ng of PCR product from each sample (both 16S and 18S rRNA) was pooled for 454 pyrosequencing.

Bioinformatics

16S and 18S Amplicons

Raw reads were demultiplexed and trimmed of adapters and low-quality sequences using an in-house script. Paired-end reads were merged using PEAR (5) with the default parameters. Merged sequences were searched for rDNA via matching for the rRNA reference covariance models using Infernal (6). Taxonomic classification of the predicted rDNA sequences was determined by conducting a blastn (7) search against the SILVA (8). A subset of the reference (SILVA) rRNA sequences was manually identified to be included in building a reference phylogenetic tree. The reference sequences were aligned with MAFFT (9). The generated multiple sequence alignment was visually inspected and manually edited and refined using JalView (10). A maximum-likelihood reference tree was inferred under the general time-reversible model with gamma-distributed rate heterogeneity and an estimated proportion of invariant sites (GTR + Γ + I), implemented in RAxML (11). The predicted metagenomic rDNA sequences were mapped onto the reference tree using pplacer (12) with the default settings. The counts of the sequences affiliated with the nodes on the reference tree were normalized to the total number of sequences from their corresponding samples. The normalized abundances are visualized as circles mapped on the reference tree such that the diameters of the circles are proportionate to the taxonomic abundances.

Metatranscriptomics

Illumina reads were processed via the RNAseq Annotation Pipeline (RAP) (13) as previously described (14) (SD3). Briefly, reads were trimmed and filtered with a length

minimum of 30 base pairs and a quality score minimum of 33. Ribopicker v.0.4.3 (15) was used to remove ribosomal RNA (rRNA) reads. CLC Genomics Workbench 9.5.3 (https://www.qiagenbioinformatics.com/) was used to assemble reads first by library, then overall. FragGeneScan (16) was used for *ab initio* ORF prediction (that is to say, gene prediction based on signal detection in the sequence itself, rather than by comparison to known genes). ORFs were screened for contamination in the form of rRNA, ITS, and primers. Organellar ORFs (those with closer homology to a known organelle gene than that of a nuclear gene in the same reference organism) were identified for separate analysis.

Ab initio ORFs were annotated for function de novo by assigning Pfams (17), TIGRfams and transmembrane tmHMMs with hmmer 3.0 (http://hmmer.org/) using an e-value threshold of $1.0e^{-4}$ as well as assigned function and taxonomic identity via BLASTP (7, 18) alignment (e-value threshold $1e^{-3}$) to a comprehensive protein database, *phyloDB*. PhyloDB includes peptides from the 410 taxa of the Marine Microbial Eukaryotic Transcriptome Sequencing Project (http://marinemicroeukaryotes.org/), as well as peptides from KEGG, GenBank, JGI, ENSEMBL, CAMERA, and various other repositories. To avoid biases introduced by taxonomically classifying ORFs based on best BLAST hit alone, a Lineage Probability Index (LPI) was calculated (19). Briefly, LPI was calculated here as a value between 0 and 1 indicating lineage commonality among the top 95-percentile of sequences based on BLAST bit-score. Reads were then mapped competitively to the full suite of *ab initio* ORFs to generate counts for each sample. Taxonomic counts were prepared by tallying reads mapped to taxonomically annotated ORFs.

Mapping to reference transcriptomes

The 22 reference transcriptomes that most commonly appeared as best hits for *ab initio* ORFs were also mapped to directly. These reference transcriptomes were sourced from the Marine Microbial Eukaryotic Transcriptome Sequencing Project (MMETSP; http://marinemicroeukaryotes.org/). Reads from the incubation experiment were aligned to both MMETSP reference contigs and ORFs with BWA-MEM(20, 21) using default parameters.

SNV detection

Single nucleotide variants (SNVs) were detected among reads mapping to *ab initio* ORFs to a maximum read depth of 8000 using samtools mpileup (22) with the –C50 parameter to reduce the effect of reads with excessive mismatches and –A to include anomalous read pairs, and bcftools call with the consensus calling method (-c). Libraries were subsampled to the depth of the lowest-coverage library to reduce coverage biases in SNV detection. Only SNVs with a quality score of at least 10, a root mean square mapping quality of at least 40, and at least 10 reads covering the call position were reported.

Clustering

Reference transcriptome ORFs were clustered together with *ab initio* ORFs (including organellar ORFs) from the five major phytoplankton groups in our data (diatoms, dinoflagellates, chlorophytes, haptophytes, and pelagophytes), to form peptide ortholog groups via the Markov Cluster Algorithm (MCL) implemented using orthoMCL (https://micans.org/mcl/) (23). Directional edge weights were defined as the ratio of pairwise- to self- BLASTP scores, and default parameters were used to assign ORFs to clusters. Clusters were assigned a consensus annotation if found to be statistically enriched in that annotation with a Fisher's exact test ($p < 0.05$). Consensus annotations must also represent at least 10% of the reads in the cluster and account for a minimum of 200 reads.

Differential expression analysis

Differential expression (DE) of taxa groups, reference transcriptome ORFs, *ab initio* ORFs, and ortholog clusters across bloom conditions was identified using edgeR version 3.16.5 (24). Read counts were normalized using the “calcNormFactors” function, which accounts for both library size and varied library composition. This function performs trimmed mean of M-values (TMM) normalization to account for a common RNA-seq effect in which the over-sampling of highly expressed genes falsely causes low-expression genes to appear downregulated. Tagwise dispersions were estimated using quantile-adjusted conditional maximum likelihood (qCML) method via the “estimateTagwiseDisp” function. P-values were computed using the “exactTest” function, which is analogous to Fisher’s exact test but uses a negative binomial distribution, and FDR-corrected using the Benjamini-Hochberg method. DE of ortholog clusters was determined by using a Fisher’s exact test ($FDR < 0.05$) in R to determine if each cluster was enriched in up- regulated ORFs, down- regulated ORFs or differentially expressed ORFs for a given taxa group. Manta plots of DE were created using the R package manta version 1.28.1.

For *ab initio* ORF DE, additional normalization strategies were implemented in order to validate the use of traditional edgeR parameters. EdgeR traditionally normalizes by distribution (TMM normalization), which assumes roughly the same number of up- and down- regulated genes across conditions. Due to the large change in biomass and physiology inherent in a bloom experiment, we sought to validate our results by normalizing to growth biomarkers including a regulator of ribosome biogenesis (Nop53), a housekeeping gene (60S ribosomal protein L7, rpl7; KOG3184) with validated stability across metazoans (25, 26), plants (27) and algae(28, 29), and a gene cluster that was highly correlated with cell counts (cluster 630, $R > 0.9$). Marker gene

normalization has previously been shown to improve the accuracy of edgeR in cases when DE is not symmetric across conditions (30, 31). Size factors for edgeR normalization were calculated in DEseq2 verison 1.14.1 (32) by providing the function *estimateSizeFactors* with the chosen markers as *controlGenes*. The size factors were then imported into edgeR using the *norm.factors* parameter of *DGEList*. In all cases, an exact test with tagwise dispersion estimation was used to determine ORFs with significantly different expression across size classes (FDR corrected $p < 0.05$). While the number of genes classified as differentially expressed varied widely across normalization strategy, gene functions with strong DE were chiefly conserved across strategies, and common markers of N and Fe stress were detected across all methods (SD4). Because these strategies yielded similar results, we chose to use the simplest, default normalization method (library normalization with “calcNormFactors”) for the differential expression data presented throughout the text.

Identification of light harvesting complex proteins

Light harvesting complex (LHC) sequences were mined from the metatranscriptomic datasets using Hidden Markov Models (HMMs; available at https://github.com/allenlab/Data_Files/blob/main/LHC_hmm). HMMs were constructed and validated using references from our in-house database, phyloDB, of complete genomes and eukaryotic transcriptomes (phyloDB 1.075 available at https://scripps.ucsd.edu/labs/aallen/data/). Sequences were trimmed to 130 amino acids, aligned using MUSCLE, and reference phylogeny was constructed using Phyml. Placement of sequencing reads from each dataset was performed using pplacer, and the final tree was drawn using in-house software for phylogenetic placement predication visualization (available at https://github.com/mccrowjp/slacTree.git). Circles indicate the relative abundance of sequences at node placement, abundance is scaled to the greatest value,

the ‘absize’ (5 here) that is a multiple used for scaling each radius size. In slacTree, final abundances are calculated using (sqrt(scaled abundance) x viewwidth x absize x 0.001). Viewwidth is given during scalable vector graphic (svg) formatting and refers to the plot size.

References

1. Garrison DL, Gibson A, Coale SL, Gowing MM, Okolodkov YB, Fritsen CH, Jeffries MO. 2005. Sea-ice microbial communities in the Ross Sea: Autumn and summer biota. Mar Ecol Prog Ser 300:39–52.
2. Lane DJ, Pace B, Olsen GJ, Stahl DA, Sogin ML, Pace NR. 1985. Rapid determination of 16S ribosomal RNA sequences for phylogenetic analyses. Proc Natl Acad Sci 82:6955–6959.
3. Herlemann DPR, Labrenz M, Jürgens K, Bertilsson S, Waniek JJ, Andersson AF. 2011. Transitions in bacterial communities along the 2000 km salinity gradient of the Baltic Sea. ISME J 5:1571–1579.
4. Stoeck T, Bass D, Nebel M, Christen R, Jones MDM, Breiner HW, Richards TA. 2010. Multiple marker parallel tag environmental DNA sequencing reveals a highly complex eukaryotic community in marine anoxic water. Mol Ecol 19:21–31.
5. Zhang J, Kobert K, Flouri T, Stamatakis A. 2014. PEAR: a fast and accurate Illumina Paired-End reAd mergeR. Bioinformatics 30:614–620.
6. Nawrocki EP, Eddy SR. 2013. Infernal 1.1: 100-fold faster RNA homology searches. Bioinformatics 29:2933–2935.
7. Altschul SF, Gish W, Miller W, Myers EW, Lipman DJ. 1990. Basic local alignment search tool. J Mol Biol 215:403–410.
8. Quast C, Pruesse E, Yilmaz P, Gerken J, Schweer T, Yarza P, Peplies J, Glöckner FO. 2013. The SILVA ribosomal RNA gene database project: Improved data processing and web-based tools. Nucleic Acids Res 41:590–596.
9. Katoh K, Standley DM. 2013. MAFFT multiple sequence alignment software version 7: improvements in performance and usability. Mol Biol Evol 30:772–780.
10. Waterhouse AM, Procter JB, Martin DMA, Clamp M, Barton GJ. 2009. Jalview Version 2-A multiple sequence alignment editor and analysis workbench. Bioinformatics 25:1189–1191.
11. Stamatakis A. 2006. RAxML-VI-HPC: maximum likelihood-based phylogenetic analyses with thousands of taxa and mixed models. Bioinformatics 22:2688–2690.
12. Matsen FA, Kodner RB, Armbrust EV. 2010. pplacer: linear time maximum-likelihood and Bayesian phylogenetic placement of sequences onto a fixed reference tree. BMC Bioinformatics 11:538.
13. Bertrand EM, McCrow JP, Moustafa A, Zheng H, McQuaid JB, Delmont TO, Post AF, Sipler RE, Spackeen JL, Xu K, Bronk DA, Hutchins DA, Allen AE. 2015. Phytoplankton-bacterial interactions mediate micronutrient colimitation at the coastal Antarctic sea ice edge. Proc Natl Acad Sci U S A 112:9938–43.
14. Kolody BC, McCrow JP, Allen LZ, Aylward FO, Fontanez KM, Moustafa A, Moniruzzaman M, Chavez FP, Scholin CA, Allen EE, Worden AZ, Delong EF, Allen AE. 2019. Diel transcriptional response of a California Current plankton microbiome to light,

low iron, and enduring viral infection. ISME J 2817–2833.
15. Schmieder R, Lim YW, Edwards R. 2012. Identification and removal of ribosomal RNA sequences from metatranscriptomes. Bioinformatics 28:433–435.
16. Rho M, Tang H, Ye Y. 2010. FragGeneScan: predicting genes in short and error-prone reads. Nucleic Acids Res 38:e191–e191.
17. Sonnhammer E, Eddy SR, Birney E, Bateman A, Durbin R. 1998. Pfam: multiple sequence alignments and HMM-profiles of protein domains. Nucleic Acids Res 26:320–322.
18. Protein BLAST: search protein databases using a protein query. https://blast.ncbi.nlm.nih.gov/Blast.cgi?PAGE=Proteins.
19. Podell S, Gaasterland T. 2007. DarkHorse: A method for genome-wide prediction of horizontal gene transfer. Genome Biol 8.
20. Bayat A, Gaëta B, Ignjatovic A, Parameswaran S. 2017. Improved VCF normalization for accurate VCF comparison. Bioinformatics 33:964–970.
21. Li H, Durbin R. 2009. Fast and accurate short read alignment with Burrows-Wheeler transform. Bioinformatics 25:1754–1760.
22. Li H, Handsaker B, Wysoker A, Fennell T, Ruan J, Homer N, Marth G, Abecasis G, Durbin R. 2009. The Sequence Alignment/Map format and SAMtools. Bioinformatics 25:2078–2079.
23. Enright AJ, Van Dongen S, Ouzounis CA. 2002. An efficient algorithm for large-scale detection of protein families. Nucleic Acids Res 30:1575–1584.
24. Robinson MD, McCarthy DJ, Smyth GK. 2009. edgeR: a Bioconductor package for differential expression analysis of digital gene expression data. Bioinformatics 26:139–140.
25. Øvergård AC, Nerland AH, Patel S. 2010. Evaluation of potential reference genes for real time RT-PCR studies in Atlantic halibut (Hippoglossus Hippoglossus L.); during development, in tissues of healthy and NNV-injected fish, and in anterior kidney leucocytes. BMC Mol Biol 11.
26. Liu Q, Lei K, Ma Q, Qiao F, Li Z-C, An L-H. 2016. Ribosomal protein L7 as a suitable reference gene for quantifying gene expression in gastropod Bellamya aeruginosa. Environ Toxicol Pharmacol 43:120–127.
27. Figueiredo A, Loureiro A, Batista D, Monteiro F, Várzea V, Pais MS, Gichuru EK, Silva MC. 2013. Validation of reference genes for normalization of qPCR gene expression data from Coffea spp. hypocotyls inoculated with Colletotrichum kahawae. BMC Res Notes 6.
28. Liu C, Wu G, Huang X, Liu S, Cong B. 2012. Validation of housekeeping genes for gene expression studies in an ice alga Chlamydomonas during freezing acclimation. Extremophiles 16:419–425.
29. Haq S, Bachvaroff TR, Place AR. 2017. Characterization of acetyl-CoA carboxylases in the basal dinoflagellate amphidinium carterae. Mar Drugs 15:1–10.
30. Evans C, Hardin J, Stoebel DM. 2018. Selecting between-sample RNA-Seq normalization methods from the perspective of their assumptions. Brief Bioinform 19:776–792.
31. McGee WA, Pimentel H, Pachter L, Wu JY. 2019. Compositional Data Analysis is necessary for simulating and analyzing RNA-Seq data. bioRxiv 564955.
32. Love MI, Huber W, Anders S. 2014. Moderated estimation of fold change and dispersion for RNA-seq data with DESeq2. Genome Biol 15:1–21.
33. Seymour JR, Amin SA, Raina JB, Stocker R. 2017. Zooming in on the phycosphere: The

ecological interface for phytoplankton-bacteria relationships. Nat Microbiol 2.
34. Bigalke A, Meyer N, Papanikolopoulou LA, Wiltshire KH, Pohnert G. 2019. The algicidal bacterium Kordia algicida shapes a natural plankton community. Appl Environ Microbiol 85:1–12.
35. Carini P, Steindler L, Beszteri S, Giovannoni SJ. 2013. Nutrient requirements for growth of the extreme oligotroph “Candidatus Pelagibacter ubique” HTCC1062 on a defined medium. ISME J 7:592–602.
