# Supplementary material for: Nitrogen and Iron Availability Drive Metabolic Remodeling and Natural Selection of Diverse Phytoplankton during Experimental Upwelling
Source: mSystems. 2022 Aug 29;7(5):e00729-22. doi: 10.1128/msystems.00729-22 (PMC9599627; doi:10.1128/msystems.00729-22)
Supplement: TABLE S1 [file msystems.00729-22-s0002.pdf]

**Abbreviation**	**Meaning**
DE	differentially expressed
DFB	desferrioxamine B (iron chelator)
DON	dissolved organic nitrogen
Fe	iron
GS	glutamine synthetase
HMM	hidden markov model
HNLC	high nutrient, low chlorophyll
ISIP	iron-starvation-induced protein
LHC	light harvesting complex
Lhcsr	stress-responsive light harvesting complex
Mn	manganese
N	nitrogen
Ni	nickel
NPQ	non-photochemical quenching
ORF	open reading frame
RdRp	RNA dependent RNA polymerase
SD	supplementary dataset
SNV	single nucleotide variant
SOD	superoxide dismutase
thiC	thiamin biosynthesis gene

Table S1 Abbreviations used in the text
